# Epigenetic mechanisms underlying prostate cancer radioresistance

**DOI:** 10.1186/s13148-021-01111-8

**Published:** 2021-06-08

**Authors:** Catarina Macedo-Silva, Rosaria Benedetti, Fortunato Ciardiello, Salvatore Cappabianca, Carmen Jerónimo, Lucia Altucci

**Affiliations:** 1 Department of Precision Medicine, University of Campania “Luigi Vanvitelli”, Vico L. De Crecchio 7, 80138 Naplei, Italy; 2 Cancer Biology and Epigenetics Group, Research Center at Portuguese Oncology Institute of Porto, F Bdg, 1st Floor, Rua Dr. António Bernardino de Almeida, 4200-072 Porto, Portugal; 3 Department of Pathology and Molecular Immunology at School of Medicine and Biomedical Sciences, University of Porto (ICBAS-UP), Porto, Portugal

**Keywords:** Epigenetics, Epidrugs, Radioresistance, DNA repair, Prostate cancer

## Abstract

Radiotherapy (RT) is one of the mainstay treatments for prostate cancer (PCa), a highly prevalent neoplasm among males worldwide. About 30% of newly diagnosed PCa patients receive RT with a curative intent. However, biochemical relapse occurs in 20–40% of advanced PCa treated with RT either alone or in combination with adjuvant-hormonal therapy. Epigenetic alterations, frequently associated with molecular variations in PCa, contribute to the acquisition of a radioresistant phenotype. Increased DNA damage repair and cell cycle deregulation decreases radio-response in PCa patients. Moreover, the interplay between epigenome and cell growth pathways is extensively described in published literature. Importantly, as the clinical pattern of PCa ranges from an indolent tumor to an aggressive disease, discovering specific targetable epigenetic molecules able to overcome and predict PCa radioresistance is urgently needed. Currently, histone-deacetylase and DNA-methyltransferase inhibitors are the most studied classes of chromatin-modifying drugs (so-called ‘epidrugs’) within cancer radiosensitization context. Nonetheless, the lack of reliable validation trials is a foremost drawback. This review summarizes the major epigenetically induced changes in radioresistant-like PCa cells and describes recently reported targeted epigenetic therapies in pre-clinical and clinical settings.

**Supplementary Information:**

The online version contains supplementary material available at 10.1186/s13148-021-01111-8.

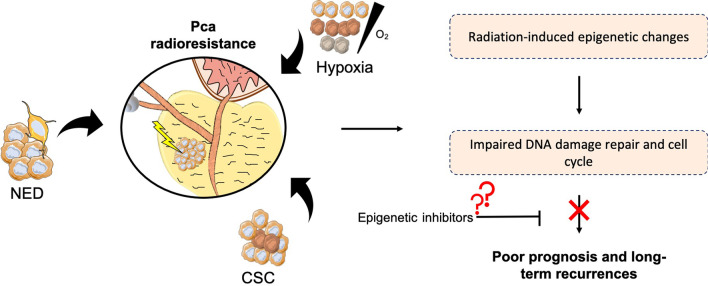

## Introduction

Prostate cancer (PCa) remains highly prevalent among males worldwide. Despite relatively low mortality rates, this malignancy is the second most common cancer in men, mostly due to the current widespread intensive prostate-specific antigen (PSA) screening [1, 2]. According to Globocan estimates, around 1.4 million new cases of PCa were diagnosed in 2020 [2].

Radiotherapy (RT) using external beam radiation therapy (EBRT) is considered a first-line standard treatment with curative intent for PCa patients, and is often performed with moderate hypofractionation therapeutic schemes using fraction sizes larger than 2 Gy delivered daily [3]. Relapse is a major clinical problem in locally advanced PCa and for both intermediate-risk and high-risk PCa (HRPC) patient’s, prognosis is significantly worse. Between 20 and 40% of patients treated with RT experience long-term recurrence within a 5-year follow-up [4]. Despite considerable efforts to develop effective therapeutic strategies, improvements in precision medicine techniques are still needed and represent the next step toward enhancing clinical management of resistance to first-line therapies.

Dynamics of ionizing radiation (IR) response is mainly associated with DNA damage pathways [5]. Hypoxic foci, PCa stem cell (PCSC) population, and neuroendocrine differentiation (NED) involved in DNA repair/apoptosis and cell cycle deregulation play a major role in radioresistance [6]. Epigenetic reprograming in PCa may also contribute to the regulation of these functional pathways [7]. Understanding how this phenotypic switch occurs and enhances radioresistance in a PCa subpopulation has been a critical research focus during the past years. Since epigenetic dysregulation is a key mechanism underlying cancer cell death escape after RT, the identification of novel epigenetic targets and new epidrugs might increment personalized clinical management [7].

In this review, we discuss the latest pre-clinical and clinical insights into the role of epigenetics in Pca radioresistance. Specifically, we focus on recent advancements in PCa’s understanding, from the molecular machineries driving radiation response based on tumor biology to epigenetic alterations involved in cell death and DNA damage pathways, all of which might impact Pca patient outcome. We also described the benefits of strategies using so-called epidrugs concurrently administered with standard RT.

## The importance of radiotherapy in prostate cancer care

Unlike other cancers, slowly proliferating tumors, such as PCa, could be highly responsive to larger radiation fraction sizes and in many cases RT constitutes the standard of care for PCa [8]. Although a meta-analysis of 25 studies including > 14.000 patients concluded that hypofractionated RT could be more effective than conventional fractions of 1.8/2 Gy [9] the major phase III trials of moderate hypofractionation did not demonstrated superiority in terms of both outcomes and toxicity (HYPRO, phase III randomized trial, ISRCTN85138529). At the same time, the hypofractionated approach, due to the added advantages of being more convenient for patients with a lower cost, has become the standard practice in the clinical management of PCa patients. Similar to normal healthy tissues with low proliferative rates, such as, kidney, lung, rectum, bladder, and brain, PCa has a long cell cycle, which confers higher damage repair efficiency and capability [8]. In fact, PCa exhibits a longer doubling time than other tumors, with a much lower fraction size (α/β ratio) of 1–2 Gy [9]. Thus, taking together radiobiological assumptions for tumor control and dose limitations of surrounding normal tissue, hypofractionation schedules in RT treatment planning might improve PCa therapeutic efficacy [8].

Recent innovative three-dimensional RT techniques, such as intensity-modulated radiation therapy (IMRT) and volumetric-modulated arc therapy (VMAT) using image-guided RT (IGRT), are overcoming previous limitations of higher dose administration schemes that resulted in late complications and tumor-related morbidities, considering the proximity of prostate gland to bladder and bowel wall [10–12]. Although conventional EBRT doses are delivered in 37–40 fractions with total delivery doses of 76–80 Gy [13], moderate to extreme hypofractionation schemes using larger daily fractions with ablative doses are reported to be more convenient in terms of costs and convenience for the patients, being non inferior in terms of both outcomes and acute/late toxicity [10].

Another approach of increasing the therapeutic ratio of RT for PCa consists of dose-escalation. *Zelensky *et al*.* in a retrospective analysis of 2551 patients with different risk categories demonstrated that biochemical disease free survival (bDFS) was significantly improved by dose escalation (above 81 Gy for the intermediate and HRPC) [14]. The FLAME trial is currently evaluating the efficacy of an integrated boost delivery of 95 Gy in multiparametric (mp) MRI-defined tumors and has shown no significant toxicities increment. Another approach of dose-escalation consists of the delivery of a high-dose rate boost of brachytherapy after, with good outcomes in terms of control of the disease [8].

Several findings support the advantages of androgen-deprivation therapies (ADT; gonadotropin-releasing hormone agonists/antagonists, abiraterone, and anti-androgens/androgen receptor [AR] antagonists) in concomitance with RT to improve overall survival rates and reduce the risk of long-term biochemical recurrences [15]. The combination of ADT with RT has definitively proven its superiority compared with RT alone followed by salvage ADT in different phase III RCTs (RTOG8610 and TROG96.01, randomized clinical trials), so that for intermediate risk PCa patients a short duration of around 6 months is advised, whereas a longer one (2–3 years) is needed for HRPC patients.

Another strategy that could improve the therapeutic efficacy in HRPC patients is the addition of chemotherapy (docetaxel) to standard RT plus ADT for a defined subset of HRPC (STAMPEDE, randomized controlled trial), although subsequent meta-analysis did not confirm the advantage in overall survival [16].

Despite the aforementioned different strategies, a significant percentage of patients progress to a castration-resistant phenotype after 2–3 years of ADT initiation, worsening the patient’s prognosis [16]. Notwithstanding advances in therapeutic management, PCa patients with high-grade tumor burden display high progression rates and consequently a greater risk of treatment failure [16]. Hence, the discovery of critical targets of unravelled molecular patterns is urgently required to predict and overcome radioresistance in this malignancy.

## Intrinsic molecular pathways involved in PCa during therapeutic radiation exposure

**Fig. 1:**
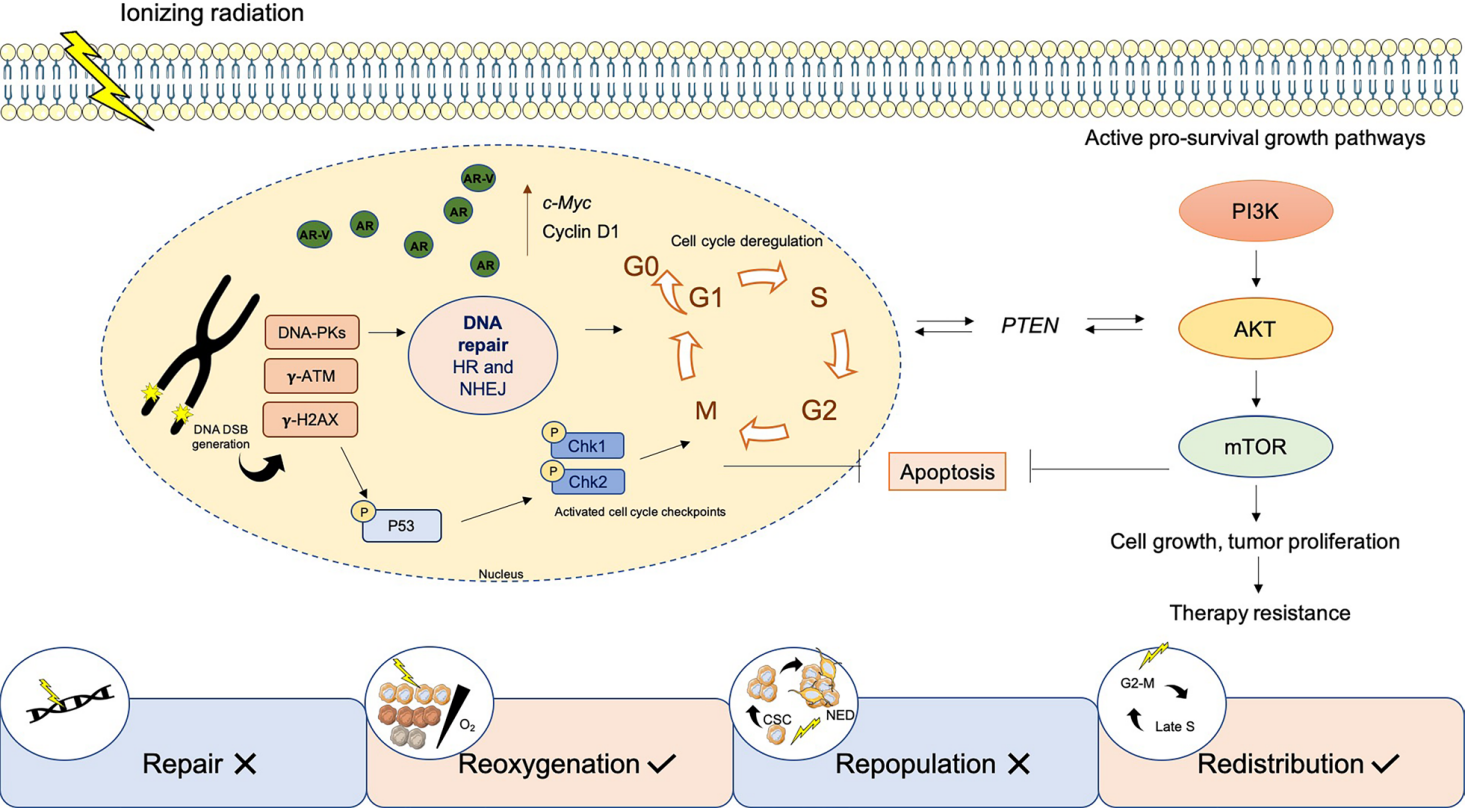
Radiation-induced radiobiological molecular pathways in PCa. Ionizing radiation exposure leads to activation of pro-survival cell growth pathways, such as PI3K/Akt/mTOR, entailing efficient DNA DSB damage repair. Specifically, radiation-induced DNA-dependent protein kinases, γ-ATM and γ-H2AX accumulation, and activation of p53 and key factors involved in cell cycle progression, sustain cell growth and tumor proliferation. Furthermore, PTEN is reported to play a role in PCa radioresistance, sustaining cell cycle arrest due to Chk1 regulation in an Akt-dependent manner. All these changes induce PCa cell growth, proliferation, apoptosis evasion, and therapy resistance. This dynamic is supported by current knowledge of the classic R’s of radiobiology, including repair of DNA damage and aggressive cell repopulation, which improve overall tumor cell survival after radiation exposure. Conversely, reoxygenation of deeper layers and cell cycle phases redistribution allows greater therapeutic efficacy.**:** AKT, protein kinase B; AR, androgen receptor; AR-V, AR variant; ATM, ataxia telangiectasia mutated; ChK1/2, checkpoint kinase 1/2; CSC, cancer stem cells; HR, homologous recombination; mTOR, mechanistic target of rapamycin kinase; NED, neuroendocrine differentiation; NHEJ, non-homologous end joining; PI3K, phosphoinositide 3-kinase; PTEN, phosphatidylinositol 3,4,5-trisphosphate 3

Considering the high degree of PCa’s histopathologic heterogeneity, RT resistance poses a major clinical challenge due to the likely development of an aggressive disease. The dynamics of successful tumor irradiation are defined by the classic R’s of radiobiology: Repair of sublethal damage, Redistribution of sensitive cell cycle phases, Repopulation, and Reoxygenation of hypoxic tumor cells (Fig. 1) [17]. As discussed above, slow proliferating tumors as PCa have a greater capacity for damage repair due to intrinsic longer cell division time [8]. All aforementioned R’s can be affected by the establishment of innovative therapeutic schemes [18]. Herein, hypofractionated approaches with higher doses per fraction might overcome the problematic of late responding tissues [18]. Latest reduced treatment time schedules should maintain elevated biological effective doses (BED) without increasing either acute or late side effects for late-responding normal tissues, which limits therapeutic designs [18, 19]. An equilibrium between minimum normal surrounding tissue damage and efficient tumor local control must be considered [18]. Using higher doses, cell toxicity and death are typically more pronounced [18]. DNA is the most critical IR target, promoting intrinsic genomic instability mainly through the generation of DNA double-strand breaks (DSBs) [20]. DNA DSB repair is mediated through two major pathways, homologous recombination (HR) and non-homologous end joining (NHEJ) [20]. Changes in the normal cell division functioning affect DNA damage repair (DDR) processes [20]. The following subsections describe the major cellular mechanisms involved in PCa radioresistance.

### DNA repair and apoptosis

The most accurate DNA repair pathway, HR, is activated during late S phase of the cell cycle, starting from the recruitment of several key proteins, such as autophosphorylated ataxia telangiectasia mutated (γ-ATM), DNA-dependent protein kinases, γ-H2AX, breast cancer gene 1/2 (BRCA1/2), poly(ADP-ribose) polymerase 1 (PARP-1), and RAD51 [21]. Defects in DDR pathways are commonly described as PCa drivers [21, 22]. Between 15 and 30% of PCa’s display DDR instability, involving the most common pathways such as mismatch repair (MMR), base-excision repair (BER), and NHEJ/HR for DSBs [23]. Specifically, DDR gene mutations were commonly found in metastatic castration-resistant PCa (CRPC) patients [24]. Impaired DNA damage repair may provide RT escape mechanisms of tumor cells. Specifically, *RAD51* – *Stat5a/b* transcriptional regulation led to efficient DSBs DNA damage repair in PCa after IR exposure [25]. Targeting this axis significantly reduced survival [25]. Involvement of phosphoinositide 3-kinase/protein kinase B/mammalian target of rapamycin (PI3K/Akt/mTOR) pro-survival signaling pathway as well as overexpression of critical cell growth and cell cycle progression factors, including *c-Myc* and *cyclin D1*, have been implicated in PCa radioresponse’s regulation [26]. Additionally, activation of cell cycle checkpoint kinases Chk1 and Chk2 in a radioresistant PCa subpopulation was found to enhance cancer stemness features, increased invasion and epithelial-mesenchymal transition (EMT) [27].

In contrast, phosphatidylinositol 3,4,5-triphosphate 3-phosphatase (PTEN), proteins involved in the caspases cascade, and apoptotic proteins are key mediators in tumor cell death [20, 28]. Nonetheless, most of these proteins are mutated or epigenetically silenced during IR exposure, resulting in apoptosis evasion [28]. Indeed, disturbances in several steps of these signaling pathways were shown to augment tumor aggressiveness, reducing RT response [20, 28]. HMG-box transcription factor 1 (*HBP1)* and *PTEN* work as tumor suppressors allowing transcription inhibition [20, 29]. Positive correlation between *HBP1* expression and RT efficacy was reported in a previous work [29]. *PTEN* is a key tumor suppressor gene (TSG) and negative Akt’s regulator found altered in PCa [20]. Intriguingly, although *PTEN* mutations are reported to contribute to tumor progression, PTEN phosphatase activity was suggested to be implicated in HR, DDR, and cell cycle arrest through Akt-dependent γ-CHK1 signaling regulation [20]. Additionally, tumor protein p53, another key dual-function protein, cell cycle and apoptosis regulator, undergoes alterations during radiation-induced DNA damage response in PCa [30, 31]. Specifically, p53 pathway upregulation was observed in PCa cells after IR exposure [30, 31]. Interestingly, p53 wild-type cells significantly reduced clonogenic capacity under RT exposure after E2F-1 transcription factor targeting [32]. These data were further supported and enriched with the combination of *MDM2* knockdown, a negative regulator of p53, resulting in γ-p53 (serine 15) enhancement [33]. Otherwise, functional phosphorylated p53 was able to prolong G1-S and G2-M cell cycle arrest, giving cells sufficient time for DDR and thus contributing to tumor cell repopulation after RT [30].

Thus, the aforementioned alterations enable tumor cells to evade mechanisms of programmed cell death, promoting recovery and repopulation between RT fractions. Direct or indirect targeting these pathways and their downstream regulators was suggested to improve PCa radiosensitization [20, 28]. Of note, 20% of PCa patients display PTEN loss-of-function mutations [34]. Driver mutations in ETS transcription factor through gene fusion played a key role in PCa [35]. ERG-ETS family member increased PARP-1 activity resulting in less DNA damage and PCa radioresistance [35]. HR- and BER-defective PCa can benefit from targeted therapy with PARP inhibitors [34], such as Olaparib and veliparib, as supported by clinical trials. Nonetheless, only pre-clinical studies shown the role of these drugs as PCa cells radiosensitizers [35–39].

### Hypoxia and PCa

Hypoxia is among the most relevant factors implicated in radioresistance and cell death escape [40, 41]. Considering the intrinsic heterogeneity of cell radiosensitivity, hypoxic foci are commonly found in both prostate hyperplasia and adenocarcinoma [42]. Theoretically, during fractionated RT, the deeper tumor layers usually farther from blood vessels become increasingly oxygenated [43]. However, for extremely hypoxic tumors with oxygen pressure (PO_2_) ≤ 1 mmHg, standard fractionated therapy was estimated to be successful in only ~ 15% of PCa patients [42]. Furthermore, hypofractionated schemes with reduced fractions might affect reoxygenation [43]. Therefore, the success rates achieved with hypofractionated schemes are not yet fully understood in PCa [42].

From a molecular point of view, hypoxia can activate a complex cellular signaling network in several tumors. The absence of oxygen was associated with hypoxia-inducible factors (HIFs) overexpression, triggering the activation of several downstream hypoxic responsive elements, such as vascular endothelial growth factor (VEGF), carbonic anhydrase IX (CAIX), and glucose transporter 1 (GLUT-1), allowing cells to adapt to the hypoxic microenvironment [44]. Severe hypoxia in the PCa niche during radiation exposure led to overall genomic instability (Fig. 1) [44]. DSBs are expected to appear after RT exposure, damaging DNA [45]. Interestingly, in hypoxic cells, along with reactive oxygen species (ROS) scavenging, the tumor microenvironment also it modulates DDR pathway [46–48]. Additionally, microenvironmental redox statement led to differential expression of NrF2 and their downstream targets allowing PCa radioresistance [49]. Conservative HR pathways and respective downstream targets were strongly deregulated both in chronic hypoxia and reoxygenation to refrain from radiation-induced cell’s death [47]. Two key HR genes, *BRCA1* and *RAD51*, as well as, the MMR genes *MLH1* and *MSH2* were also downregulated in hypoxia [45, 47]. This genetic instability leads to errors through cell cycle checkpoints, maintaining tumor progression at a high mutational rate [47]. These include genetic aberrations such as point mutations or gene amplification [47]. Indeed, highly hypoxic PCa presents a stout reduction of PTEN transcription and increased mutational burden [50].

Furthermore, PI3K/Akt/mTOR and mitogen-activated protein kinase/extracellular regulated kinase (MAPK/ERK) are critical cell signaling pathways activated during hypoxia stabilization in PCa to regulate HIF synthesis [44]. *VEGF* expression was also reported to be induced after PI3K activation in a HIF-dependent manner and via Notch cascade signaling activation, even in oxygen-independent conditions, due to *VHL*, *p53*, and *PTEN* mutations or loss of function [44, 51]. Additionally, several key DDR-related genes, including *ATM/ATR*, *Chk1/2*, γ-H2AX, and Ku70/80, were upregulated in hypoxic cells, mainly through NHEJ repair pathway [47].

### PCa stemness

PCa RT responsiveness was also regulated by a subpopulation of cells with stemness properties within tumor bulk [52]. Indeed, these cancer stem cells (CSCs), also known as tumor-initiating cells, are AR-independent and commonly maintained into the hypoxic tumor microenvironment [52–54]. These CD44^+^ and CD133^+^ cells persist after RT, promoting tumor progression and early biochemical recurrences [55]. Moreover, its avoided apoptosis and other radioresistant associated features (Fig. 1) [52]. The PCSCs specific-associated pluripotency genes, *SOX2*, *OCT3/4*, *KL-F4*, *c-Myc*, *Nanog,* and *Snail* were shown to be useful to identify recurrent patients after RT [27]. Indeed, these pluripotency-associated markers and EMT-associated molecules are induced by the HIF signaling network [56]. Furthermore, PI3K/Akt/mTOR and MAPK/ERK are commonly activated signaling pathways associated with PCSC growth and therapy evasion [57].

CSCs are distinguished by a constant quiescent state of the cell cycle (G0/G1), contrasting with the rapid cell division required RT effectiveness. Of note, defects in DDR pathways have been often observed in PCSCs [55]. An imbalance between low levels of intracellular ROS-produced DNA damage and higher DDR efficiency through γ-H2AX, Ku70, and Ku80 was also described in PCSCs after RT [55]. Cell cycle and DDR defects implicate in PCSCs radioresistance. Remarkably, *Chk1* Knockdown prevented G2-M radiation-induced cell cycle arrest, decreasing DDR, while inducing apoptosis in a CD133^+^/CD44^+^ subpopulation [58].

### PCa neuroendocrine differentiation

NED is a frequent transitory event in CRPC associated with treatment failure [59, 60]. Currently, NED is accepted to be the most aggressive clinical variant form of PCa [59]. Approximately 1% of primary PCa is classified as NED tumors at diagnosis, whereas about 30% of advanced PCa patients display neuroendocrine transitory foci [61, 62]. This phenotype is characterized by the loss of characteristic markers of the prostate gland, such as AR and PSA [63]. Instead, AR-null PCa cells display increased NED markers’expression [64]. AR-dependent signaling is compensated in neuroendocrine PCa (NEPC) through enhanced activity of cell-sustaining growth factors, such as fibroblast growth factor and MAPK [64]. Consequently, as NEPC’s biology is not fully understood, effective treatment options are very limited. Nevertheless, cisplatin-based chemotherapeutic is routinely used to reduce tumor burden in these patients [62].

Radiation exposure was also reported to induce NEPC differentiation [65]. Additionally, NE-like cells transdifferentiation upon fractionated radiation exposure have been linked with the dynamic regulation of transcription factors, such as, AMP-response element binding protein (CREB) and activating transcription factor 2 (ATF2), at nuclear (active form) and cytoplasmatic location (inactive form), respectively [66]. Furthermore, CREB inhibition led to the blockage of IR-induced NED and radiosensitize PCa cells inducing cell death [67]. Similarly to PCSCs, NEPC cells are quiescent [68], also suggesting great ability to DDR after radiation exposure. Globally, NEPC cells are molecularly characterized by *N-Myc* overexpression, which drives the transcriptional enrichment of HR DDR and stress-related genes, such as *BRCA1*, *PARP1/2*, RecQ-mediated genome instability 2 (*RMI2)*, and DNA topoisomerase II binding protein 1 (*TOPBP1)* [59]. Furthermore, *TP53*, *PTEN*, and retinoblastoma (*RB1)* loss of function were found in NEPC tumors [69]. This leads to blockage of pathways related to cell growth and proliferation inhibition, including interleukin 8-mediated IL-8/CXCR2/p53-pathway and further increasing aggressive properties of neuroendocrine malignancies [68]. Moreover, tumor plasticity plays a central role in NED due to the enhancement of EMT-induced pathways, with the activation of ZEB1/2, Snail, Slug, Twist, and N-cadherin [69]. These tumor cells have the ability to adapt, bypassing barriers, and becoming increasingly aggressive. Previous studies showed that both ADT and RT promote hypoxia-related CSC, strongly contributing to heterogeneous disease and consequently RT failure [70, 71].

Overall, lethal NEPC represents a hurdle to both RT/ADT therapy. Hence, the study of these patients’ epigenetic profile might allow for the identification of RT responders and non-responders markers, considering the previously mentioned molecular pathways involved in radioresistance.

## Epigenetics in PCa: a brief overview

**Fig. 2:**
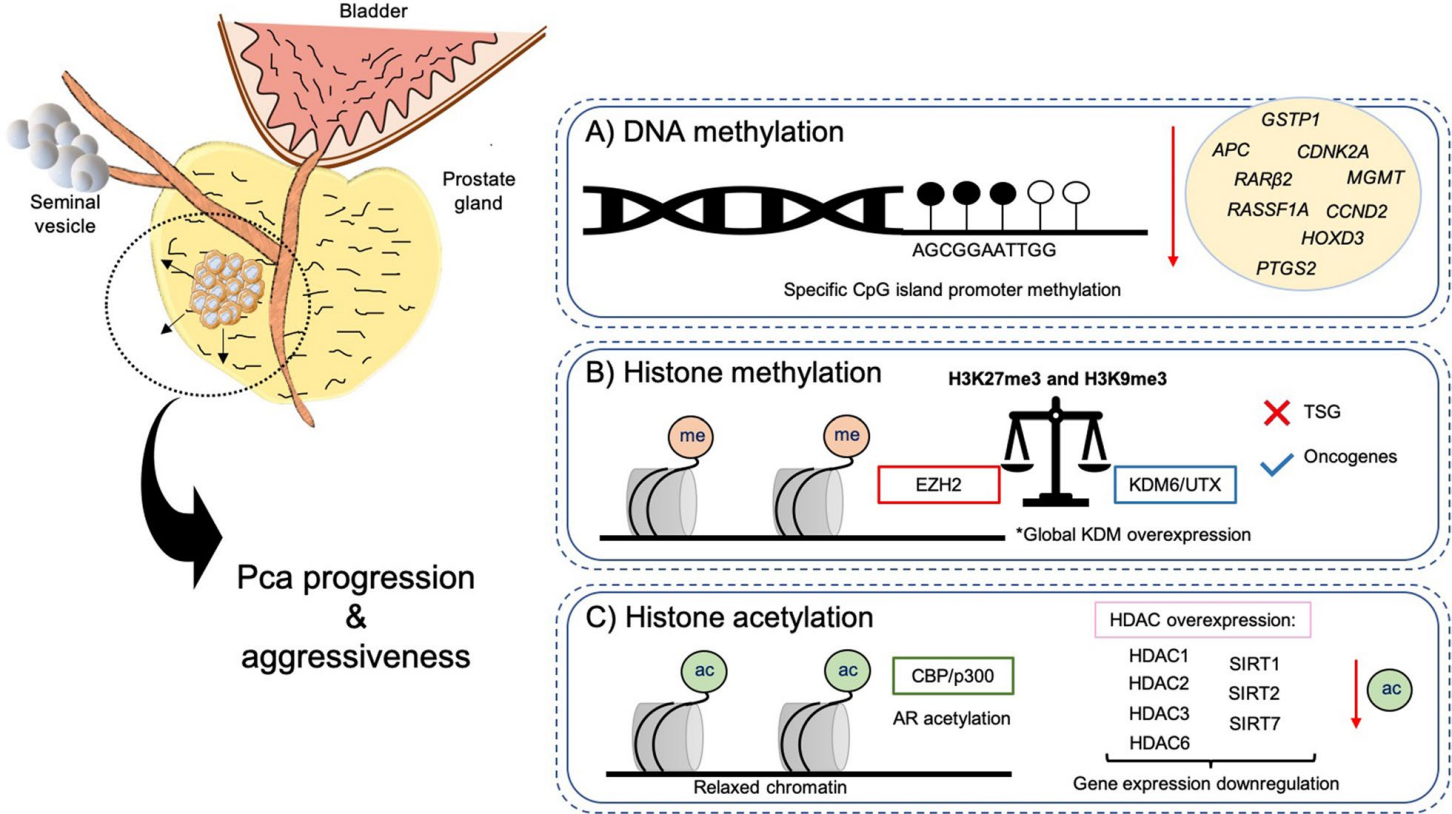
Epigenetic landscape in PCa. Aberrant DNA methylation (**A**) and histone post-translational modifications (**B**, **C**) lead to overall PCa progression and aggressiveness due to uncontrolled gene transcription signature. Black filled circles represent methylated sites (**A**). ac, acetylation; *APC*, adenomatous polyposis coli; AR, androgen receptor; CBP, CREB-binding protein; *CCND2*, cyclin D2; *CDNK2A*, cyclin-dependent kinase inhibitor 2A; CpG, cytosine/guanine enriched sites; EZH2, enhancer of zeste homolog 2; *GSTP1*, glutathione S-transferase Pi 1; HDAC, histone deacetylase; *HOXD3*, homeobox protein Hox-D3; KDM, lysine demethylase; me, methylation; *MGMT*, O^6^-methylguanine-DNA methyltransferase; PCa, prostate cancer; *PTGS2*, prostaglandin-endoperoxide synthase 2; *RARβ2*, retinoic acid receptor beta 2; *RASSF1A*, Ras association domain family member 1; SIRT, sirtuin; TGS, tumor suppressor genes

Epigenetic alterations induce reversible and heritable changes promoting differences in gene expression without changing DNA sequence [72]. They are generally classified as a constitutive hallmark of cancer, due to the combined action of oncogene activation and TSG knockdown [72]. DNA methylation, covalent histone-modifications, histone-variants and chromatin remodeling complexes are commonly accepted epigenetic mechanisms [72]. Nonetheless, PCa displays complex epigenetic landscape deregulation associated with changes in cell growth pathways and overall tumor progression (Fig. 2) [72].

### DNA methylation

DNA methylation is the major epigenetic mechanism described in cancer [73]. Differential methylation levels among distal and genic regions are key dynamic drivers of PCa tumorigenesis and progression [74]. This process involves the addition of a methyl group to 5-methylcytosine (5mC), usually at the promoter region, in CpG-enriched islands, to promote gene silencing [72]. This reaction is catalyzed by DNA methyltransferases (DNMTs), including DNMT1, often associated with DNA methylation maintenance, and DNMT3a and DNMT3b, responsible for de novo methylation [72]. In contrast, DNA methylation is reversed by ten-eleven translocation (TET) enzymes [75]. In PCa, global DNA hypomethylation has been widely reported [76, 77]. Additionally, long interspersed nuclear element-1 (*LINE-1*) a gene frequently silenced in the normal human genome, is re-expressed in PCa cells [77]. Conversely, hypermethylated foci in specific TSG promoter regions were associated with gene silencing (Fig. 2A) [72]. DNMTs, particularly DNMT3a and DNMT3b, were highly expressed in PCa and associated with tumor progression [78]. Assessment of DNA- methylation based panels along with PSA screening demonstrated a high value for prediction and/or recurrence detection in PCa patients [79]. Adenomatous polyposis coli (*APC)* is a common transcriptionally repressed TSG associated with PCa progression [80]. Additionally, well-known specific TSGs and genomic stability regulators such as retinoic acid receptor beta2 (*RARβ2*), cyclin-dependent kinase inhibitor2A (*CDNK2A*), Ras association domain family member1A (*RASSF1A*), homeobox gene D3 (*HOXD3*), O^6^-methylguanine DNA methyltransferase (*MGMT*)*,* cyclin2 (*CCND2*)*,* prostaglandin-endoperoxide synthase 2 (*PTGS2*)*,* and glutathione S-transferase Pi1 (*GSTP1*) were also commonly silenced by promoter methylation in PCa (Fig. 2A).

### Chromatin remodeling modifiers

Histone post-translational modifications (PTMs) are enzymatic modifications of proteins. These reactions are *written*/established by histone methyltransferases (KMTs) and histone acetyltransferases (HATs) and *erased*/removed by histone demethylases (KDMs) and histone deacetylases (HDACs). Tumor plasticity and epigenetic dynamics may lead to tumor cells’ aggressive phenotypes, like those found in CSC markers, including, tumor growth and proliferation, metastasis, and resistance to therapy [81].

#### Histone methylation

Deregulation of KMTs and KDMs was observed in PCa cells and was associated with cancer cell proliferation [82]. These changes generally have a pleiotropic effect that can either lead to gene transcription-repression or -activation [72]. The KMT enhancer of zeste homolog 2 (EZH2) is strongly overexpressed in PCa cells, including aggressive NEPC cells, and is generally linked to transcriptional repression through trimethylation of lysine 27 on histone 3 (H3K27me3) [83]. A member of the Jumonji C-domain (JmjC) 2-oxoglutarate-dependent dioxygenase KDM superfamily, JMJD3/UTX/KDM6, that acts as a transcriptional activator is also deregulated in PCa (Fig. 2C) [84]. This tight balance between methylation and demethylation of histone 3 and the downstream impact in specific pro-tumorigenic or anti-neoplastic gene targets is a major challenge in translational and precision medicine research, meaning that specific epidrugs targeting either EZH2 or KDM6 can elicit antagonistic effects in tumor cells, depending on their context and environment [84]. In PCa samples, H3K27me3 mark was reported to be enriched in several specific promoter regions of TSGs, such as *FBXO11*, *ING3*, *and RKIP*, as well as other tumor regulatory genes [85]. In contrast, other studies found that KDM6 enhanced activity mediating transcriptional activation of specific target genes involved in key PCa carcinogenic pathways, including AR signaling [84]. Moreover, KMTs downregulation, namely SUV39H1 (KMT1A) results in lower levels of another repressive mark, H3K9me3, in PCa cells [86]. Increased expression of several KDMs including LSD1 (KDM1A), a lysine-specific demethylase, and JmjC-KDMs as JMJD1A/KDM3A, JMJD1B/KDM3B, JMJD2A/KDM4A, JMJD2C/KDM4C, JARID1B/KDM5B, and PHF8/KDM7B was also reported in PCa, resulting in higher PCa proliferation, migration, and invasion (Fig. 2C) [87].

#### Histone acetylation

Histone acetylation is another widely studied histone modification in PCa. Unlike histone methylation, acetylation is usually associated with transcriptional activation due to DNA molecule relaxing by histone charge neutralization. Co-activator complex proteins with a conserved HAT domain, such as p300/CREB binding protein (CBP), play a key role in the progression of AR-dependent PCa cells through AR acetylation (Fig. 2D) [88]. Hyperacetylation of histone 3 lysine residues 9, 14, and 18 (H3K9ac, H3K14ac, H3K18ac) induces castration-resistant progression in PCa cells via p300 activity [89]. Additionally, higher levels of acetylated histone 4 lysine 16 (H4K16ac) in PCa cells was reported to induce transcriptional activation of pro-inflammatory genes, such as tumor necrosis factor alpha (TNF-α) and nuclear factor kappa-light-chain-enhancer of activated B cells (NF-kB) [90].

Furthermore, HDACs are a large family of chromatin remodelers comprising four major classes (I, II, III, and IV) regulating both transcription factors and histone deacetylation [81]. Class III HDACs are a particular class of sirtuins (SIRTs) with a NAD^+^-dependent catalytic activity mechanism, whereas other classes are zinc-dependent. Histone deacetylation is a common PTM in PCa generally leading to transcriptional repression of specific genes [81]. HDACs’ overexpression, including HDAC1, HDAC2, and HDAC3, was tightly associated with PCa progression and aggressiveness (Fig. 2D) [91]. Specifically, HDAC1 activity induced *Yan Yang 1*(*YY1)*-mediated repression of XIAP-associated factor 1 (*XAF1*) in PCa cells [92]. Furthermore, HDAC2 was named a good candidate PCa prognostic biomarker [91]. The combined effect of both mechanisms, such as increased H3K27me3 and decreased H3K9ac, induced tumor suppressor *TIMP3* inhibition*,* in PCa cells [93]. Increased HDAC activity was associated with *ERG* expression, which inhibits HATs activity in PCa cells [94]. Interestingly, HDAC6 (a class IIb HDAC) regulates AR protein stabilization in CRPC, through Hsp90 transcription factor substrate (Fig. 2D) [95]. Conversely, HDAC4 (a class IIa HDAC) an endogenous regulator, binds with AR, inhibiting its activity in AR-dependent PCa cells through SUMOylation, another epigenetic remodeling mechanism [96]. SIRTs are also overexpressed in PCa cells. Specifically, SIRT7 upregulation lead to PCa progression, increased cell migration, and invasion (Fig. 2D) [97]. These findings support the clinical value of using these targets as predictive biomarkers in advanced PCa patients. Further discussion about available drugs for these targets will be done in the next section of radiosensitizing strategies.

## Radiation and epigenetic dynamics interactions

**Fig. 3:**
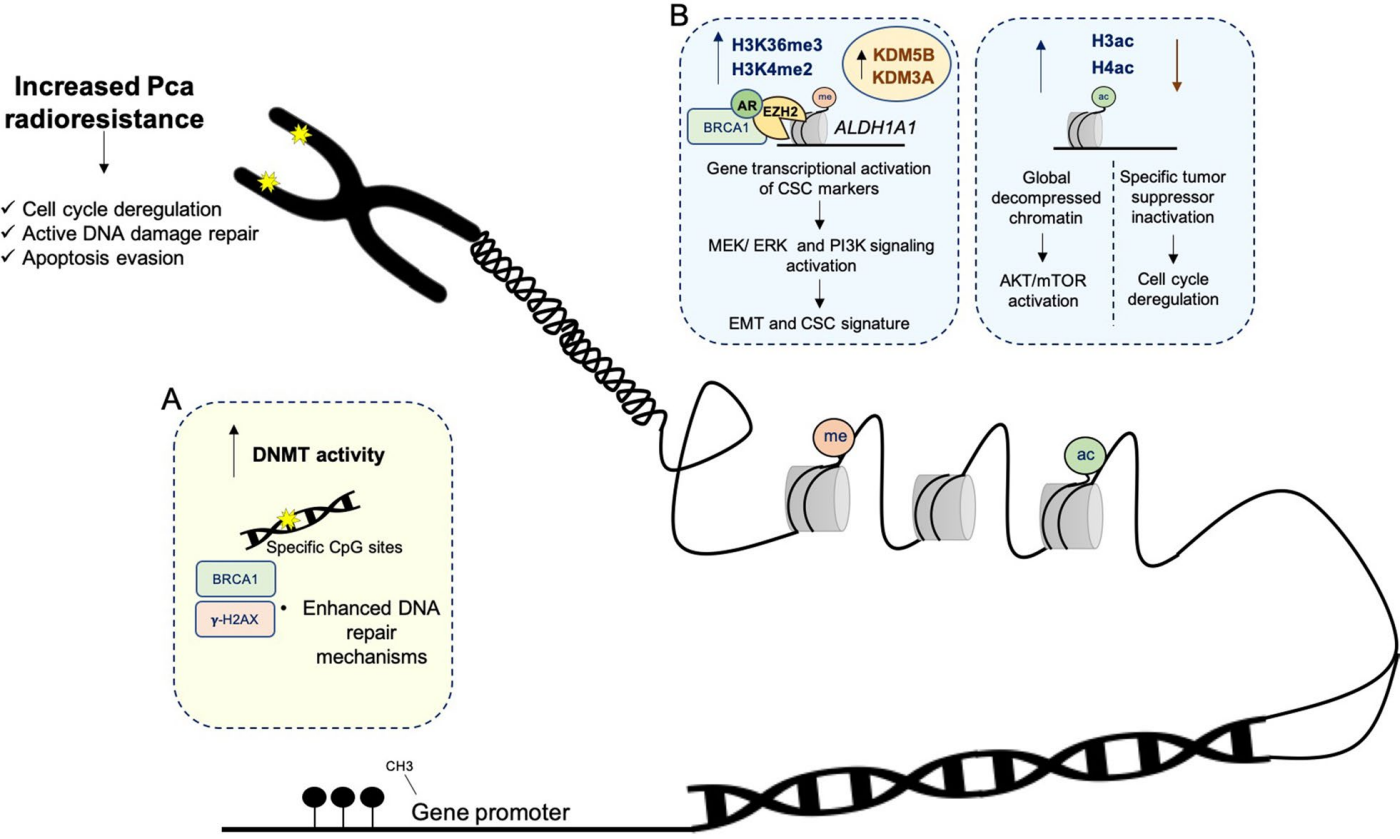
Radiation-induced epigenetic reprograming in PCa. Epigenetic mechanism regulation plays a key role in PCa radiation response, contributing to cell cycle deregulation, active DNA damage repair, and apoptosis evasion. **A** Aberrant gene expression is mediated by high DNMT activity at specific CpG sites. This mechanism allows DNA damage repair efficacy and apoptosis evasion. **B** Histone post-translational modifications are able to modulate cell growth, CSC and EMT gene signature, and cell cycle deregulation. Uncontrolled PCa cell proliferation is maintained by an imbalance between repressive and activating markers. Black filled circles represent methylated sites. ALDH1A1, aldehyde dehydrogenase 1 family member A1; AR, androgen receptor; AKT, protein kinase B; BRCA1, breast cancer type 1; CSC, cancer stem cells; DNMT, DNA methyltransferase; EMT, epithelial-mesenchymal transition; ERK, extracellular regulated kinase; EZH2, enhancer of zeste homolog 2; HDAC, histone deacetylase; MEK, mitogen-activated protein kinase; mTOR, mechanistic target of rapamycin kinase; PI3K, phosphoinositide 3-kinase

Although epigenetic regulation has been implicated in cellular radiation response control of PCa [98], convincing clinical evidence and validation of these findings are lacking. As discussed above, cancer cell death is the major goal in radiation-based therapy. Importantly, targeting DNA repair and cell cycle regulatory pathways might overcome PCa radioresistance [99]. Overall, radiation was demonstrated to induce chromosomal instability, mainly through specific methylation patterns and a wide range of histone PTMs [100]. Epigenomic modifications are involved in cell growth pathways and radioresistant signatures regulation. Concurrently, radiotherapeutic treatment was also found to cause epigenetic changes in PCa cells [101]. Notably, although global DNA hypomethylation has been considered a hallmark of most cancers (by contributing to general genomic instability), this is commonly accompanied by increased DNA methylation levels at specific CpG sites and gene promoters (namely those belonging to tumor suppressor genes), as observed after exposure to IR (Fig. 3A) [102–104]. These changes influenced the recruitment of DSB repair agents, such as γ-H2AX and BRCA1, for an efficient damage response in PC-3, a radioresistant PCa cell line (Fig. 3A) [102]. Additionally, PCSC growth was stimulated after RT exposure through specific epigenetic modulation [7]. Accumulation of H3K36me3 at aldehyde dehydrogenase 1A (*ALDH1A1*) promoter region, a CSC-related marker, along with EZH2 overexpression, associated with radiation-induced resistance in PCa (Fig. 3B) [7]. Accordingly, enhanced EZH2 activity cooperates with BRCA1, maintaining CSC signature and PCa radioresistance [105]. Furthermore, EZH2 sustained MEK/ERK signaling pathway activation during EMT, allowing PCa proliferation and invasiveness (Fig. 3B) [106].

As previously described, PI3K and androgens are key determinants of PCa signaling pathways, sustaining tumor growth after cell death-based treatments. Uncontrolled cell cycle proliferation and stimulated growth pathways are determined by epigenetic mechanisms [81]. Hence, increased androgen driven H3K4me2 levels activate PI3K signaling pathway, resulting in CRPC progression (Fig. 3B) [107]. Androgen-mediated effects were previously associated with a relaxed chromatin structure due to higher histone acetylation levels [108]. Both H3 and H4 acetylation activate Akt/mTOR, increasing PCa progression, and therapy resistance (Fig. 3B) [108]. Conversely, increased HDAC activity was linked to specific tumor suppressor inactivation in PCa (Fig. 3B) [91]. Moreover, several HDAC inhibitors (HDACi) were described as radiosensitizers, affecting both DSB repair machinery and cell cycle controlling pathways [109]. In terms of histone methylation, inactive KDM5D induced aberrant and faster cell division, activating DDR-related pathways in PCa, such as enhanced ATR kinase activity, conferring a more aggressive phenotype (Fig. 3B) [110]. Conversely, KDM5B overexpression led to a radioresistant phenotype in non-small cell lung cancer and PCa cell lines (Fig. 3B) [111]. Herein, clinical samples of non-small cell lung cancer showed lower RT response rates when higher levels of KDM5B were detected [111]. KDM3A, with substrate selectivity for H3K9me1/2, also induced aberrant DDR activation in radioresistant PCa [112]. Reversion of these epigenetic changes affected the recruitment of DSB repair molecules such as γ-H2AX and ATM, increasing tumor radiosensitivity [111]. Furthermore, a key regulatory cell cycle component, cyclin D2, is a candidate target of SMYD3 (KMT that specifically catalyzes transcriptional suppressive trimethylation of H4K20) [113]. This interplay, leads to deregulated cell cycle pathways and aberrant mitosis, resulting in uncontrolled PCa cell progression [113].

## Strategies for radiosensitizing tumor cells with well-known epigenetic targeting drugs

The development of epigenetic compounds with radiosensitizing properties rationalizes the use of these drugs in a clinical context. Nevertheless, the demonstration of solid effectiveness of epigenetic-based radiosensitization in PCa is still lacking. Although a wide range of HDACi tested in several cancer models including PCa showed impressive anticancer properties, the specific effect of these epidrugs in cells after IR exposure remains elusive and do not progress after pre-clinical level. To date, only few clinical trials investigated the radiosensitizing effect of these inhibitors are summarized in Table 1. Remarkably, the major drawback of these studies are the toxicity effects. Additionally, larger randomized trials to corroborate the tolerability to the drugs and to evaluate the efficacy in the local control are needed. In fact, most of these clinical trials included a reduced number of participants who had completed the protocol. Furthermore, especially in relation to HDACi, to improve their current adoption into the clinical practice, it would be pertinent and helpful the development of newer, more specific HDACi or other epidrugs, as discussed previous. These inhibitors only target global histone acetylation and not specific lysine sites.

**Table 1:** Epigenetic clinical trials with well-known epidrugs to overcome cancer radioresistance

Drug	Clinical trials	Target	Cancer type	Patients	Doses	OS rates	Year of publication	References	Study ID
SAHA	Phase I	HDACi	Brain metastasis	4 completed study protocol	400 mg/day with 37.5 Gy (2.5 Gy/fr.) over 5 weeks	36 weeks	2014	[119]	NCT01600742
SAHA	Phase I	HDACi	NSCLC brain metastasis	12 completed study protocol	200–400 mg/day for 14 days with SD of SRS at day 3	13 months	2017	[120]	NCT00946673
SAHA	Phase I/II	HDACi	Glioblastoma	Phase I: 15 Phase II: 107	300 or 400 mg/day with Std TMZ + RT	55.1%, 15 months FU	2018	[121]	NCT00731731
SAHA	Phase I	HDACi	Advanced head and neck SCC	26 completed study protocol	100–400 mg, 3 × weekly with concurrent CRT	96.2% CR at 33.8 months FU	2019	[122]	NCT01064921
VPA	Phase II	HDACi	Glioblastoma	37 completed study protocol	25 mg/kg oral divided in 2 dd concurrent with RT and TMZ	97%, 86%, and 65% at 6, 12, and 24 months FU, respectively	2015	[123]	NIHMS686154
VPA + Hydralazine	Phase III	HDACi + DNMTi	Stage III cervical cancer	18 completed study protocol	182 mg or 83 mg of hydralazine and 30 mg/kg VPA plus TCD of 85 Gy	NS	2010	[124]	NCT02446652
LBH-589	Phase I	HDACi	Stage III NSCLC	9 with pRT	20, 30, 45 mg twice/week, with pRT or rCRT	9 months	2015	[125]	NA
LBH-589	Phase I	HDACi	High grade gliomas	12 completed study protocol	10, 20, 30 mg/day, with 30–35 Gy (10 fr.)	7.8; 6.1, and 16.1 for each drug concentration, respectively	2016	[126]	NCT01324635
LBH-589	Phase I	HDACi	HNC, PCa and Esophageal cancer	7 completed study protocol	NS	NS	2017	NP	NCT00670553

CR, complete response; CRT, chemoradiotherapy; DNMTi, DNA methyltransferase inhibitors; FU, follow-up; Fr., fraction; HDACi, histone deacetylase inhibitors; HNC, Head and neck cancer; NS, not specified; OS, overall survival; RT, radiotherapy; TMZ, temozolomide; VPA, Valproic acid; pRT, palliative radiotherapy; PCa, prostate cancer; NA, not available; NP, not published; NS, not specified; NSCLC, non-small cell lung cancer; SCC, squamous cell carcinoma; SD, standard dose; SRS, stereotactic radiosurgery

**Fig. 4:**
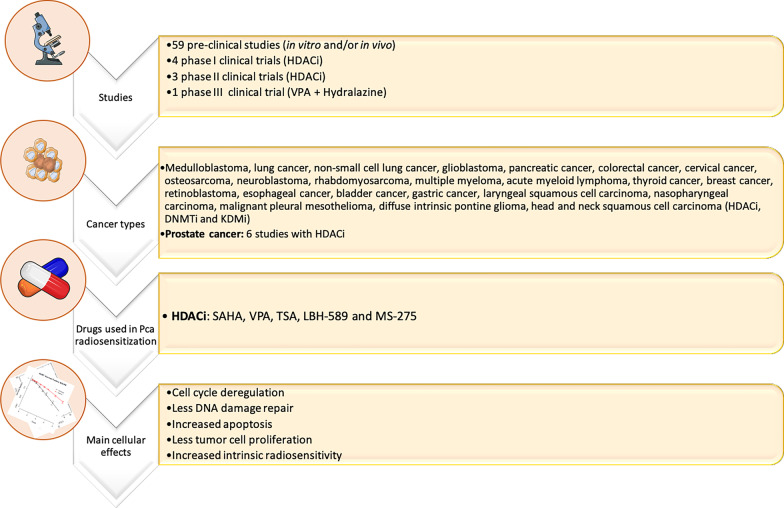
Epigenetic targeting strategies to improve the clinical management of radioresistance. 59 pre-clinical studies have investigated a wide range of HDACi, DNMTi, and KDMi for radiosensitization purposes. Only 6 studies were conducted in PCa models. A further 8 clinical trials (Phase I, II, and III) are evaluating the use of HDACi and DNMTi in several cancer types in combination with conventional RT schemes. Overall, the use of these epidrugs results in cytotoxic effects promoting tumor cell death. DNMTi, DNA methyltransferase inhibitor; HDACi, histone deacetylase inhibitor; KDMi, histone lysine demethylase inhibitor. For additional information please access Additional file 1

Vorinostat, an FDA-approved HDACi (also known as SAHA) displays a potential radiosensitizer effect in several cancers (Fig. 4 and Additional file 1). Specifically, vorinostat improved radiosensitivity in three PCa cell lines with different radiation response patterns, both in normoxia and hypoxia conditions [114]. The radiosensitizer use of vorinostat decreased cell survival fraction and significantly increased G2/M cell fraction, along with specific *HIF-1α* and *TP53* target downregulation [114]. Several clinical trials have reported considerable benefits of vorinostat in many cancer types subjected to IR (Table 1). Valproic acid (VPA), a common anti-epileptic drug, also a well-known HDACi, radiosensitize several cancers undergoing fractionated RT (Fig. 4 and Additional file 1). Low VPA concentrations (50 μM) sensitized PCa cells to IR through p53 acetylation stabilization and enhanced apoptosis (Fig. 4 and Additional file 1). Indeed, several reported studies corroborate these findings in other cancers (Fig. 4 and Additional file 1, Table 1). Trichostatin A (TSA), another HDACi, sensitized a PCSCs radioresistant fraction and depicted similar effect in other cancer models, showing increased radiation-induced DNA damage (Fig. 4). In the same vein, panobinostat (LBH-589), pan-HDACi, was able to increase PCa radiosensitivity as well as in other cancer types (Fig. 4 and Additional file 1, Table 1). The radiosensitizing effect of entinostat (MS-275) was studied in vitro in PCa and glioma cells (Fig. 4 and Additional file 1). Herein, along with irradiation, entinostat increased tumor cell apoptosis , as well as, radiosensitivity (Fig. 4 and Additional file 1). Although other well-known HDACi’s, such as FK228, CBHA, sodium butyrate, TMP195, and mocetinostat, were reported to exhibit a potential radiosensitizing effect by affecting DNA repair pathways (HR and NHEJ) in several malignancies, studies are lacking for PCa (Fig. 4 and Additional file 1).

DNA hypomethylating agents, DNMT inhibitors (DNMTi), also play a role in tumor radiosensitization. Specifically, the use of HDACi in combination with DNMTi showed a cumulative effect on cancer radiosensitization (Table 1, reporting combinatorial radiosensitization clinical trials in PCa and other cancers). 5-aza-2’-deoxycytidine (DAC) is the most commonly studied DNMTi (Fig. 4 and Additional file 1). Nonetheless, no reports are still available about PCa radiosensitization by these epidrugs (Fig. 4 and Additional file 1). Concerning histone methylation, JmjC-KDMs and LDS/KDM1A inhibitors are two well-known epigenetic drugs, with a limited number of studies about cancer radiosensitization. GSK-J4 is the most studied JmjC-KDM inhibitor, with KDM6/UTX specificity and cancer radiosensitivity effect (Fig. 4 and Additional file 1). Another JmjC-KDM inhibitor with higher specificity for KDM5 subfamily inhibition, JIB-04, was able to enhanced radiation response of lung squamous cell carcinoma cells [111]. Despite extensive knowledge of KDMs’ implication in hypoxia and tumor aggressiveness, as discussed in fifth section, few recent pre-clinical studies provided evidence that KDM activity inhibition may associate with tumor radiosensitization. Remarkably, LSD1/KDM1A has been highlighted as an important chromatin and gene transcriptional modulator in PCa [115]. LSD1 inhibitors are emerging as successful drugs with important role in PCa progression blockage, as well as, CRPC growth suppression [115]. However, there are a lack of approved inhibitors in radiation field with clinical validation. Of note, previous in vitro reports shown LSD1 transient recruitment to DNA damage allowing the activation of HR DNA repair machinery, such as 53BP1 and BRCA1 [116]. Herein, mechanistic studies with LSD1 knockdown significantly enhanced radiosensitivity [116]. Hence, LSD1 seems to be an interesting target to use in future as a tumor radiosensitizer, also for PCa. Additionally, BRD4, a bromodomain family-member with essential role as chromatin remodeler has been reported as a synergic interactor of LSD1 in CRPC [117]. Furthermore, BRD4 has a critical role for NHEJ DNA damage repair pathway and a low prognostic value for PCa patients submitted to RT [118]. In fact, according to the aforementioned clinical trials using epigenetic drugs, most of the inhibitors used are broad range effectors. The lack of specific targeting drugs is a critical drawback in the research field. Overall, further insights in the discovery of promising targetable epigenetic enzymes are urgently needed. For instance, as previously discussed in fifth section, KDM3A [112] and SMYD3 [113] were reported with a critical role as DNA damage and cell cycle mediators in PCa. The expression of these enzymes is commonly associated with poor PCa prognosis. However, until now there are no further validation with specific targeting epigenetic drugs.

## Conclusions and future perspectives

Although PCa mostly constitutes a rather indolent malignancy, in a non-negligible percentage of cases can progress to highly aggressive disease. Advancements in translational research and innovative precision medicine techniques are urgently needed to determine the optimal clinical management based on intrinsic tumor biology. As many PCa patients are primarily treated with standard fractionated RT schemes, the development of novel adjuvant targeted strategies is imperative to overcome high recurrence rates of 20–40% in this subgroup of PCa patients. Epigenetic targeting might be the key to improving and extending survival of PCa patients. Indeed, epigenetically-inducible changes in DNA repair pathways and cell cycle play a critical role in response to IR. Cellular dynamics at epigenetic level led to overall tumor aggressiveness, increasing cell proliferation, invasion, and migration. Thus, the identification of specific key epigenetic targets and the use of epidrugs, such as inhibitors of DNMTs, HDACs, or KDMs, might radiosensitize tumor cells, increasing patients’ response to therapy and overall survival. Nevertheless, other studies are needed to further support these epidrugs as a novel treatment that should be used in combination with standard RT to improve PCa patients’ outcome.

## Supplementary Information

**Additional file 1.** Complementary pre-clinical studies for tumor radiosensitization.

## Data Availability

Not applicable.

## Abbreviations

RT: Radiotherapy
PCa: Prostate cancer
PSA: Prostate specific antigen
EBRT: External beam radiation therapy
HRPC: High risk prostate cancer
IR: Ionizing radiation
PCSC: Prostate cancer stem cells
NED: Neuroendocrine differentiation
IMRT: Intensity modulated radiotherapy
VMAT: Volumetric-modulated arc therapy
IGRT: Image-guided radiotherapy
bDFS: Biochemical disease-free survival
ADT: Androgen deprivation therapy
DSB: Double strand breaks
DDR: DNA damage repair
y-ATM: Autophosphorylated ataxia telangiectasia mutated
BRCA: Breast cancer gene 1/2
PARP: Poly(ADP-ribose) polymerase 1
BER: Base excision repair
NHEJ: Non homologous end-joining
HR: Homologous recombination
CRPC: Castration resistant prostate cancer
EMT: Epithelial mesenchymal transition
HIF: Hypoxia-inducible factor
VEGF: Vascular endothelial growth factor
CAIX: Carbonic anhydrase IX
GLUT-1: Glucose transporter 1
ROS: Reactive oxygen species
PTEN: Phosphatidylinositol 3,4,5-triphosphate 3-phosphatase
CSC: Cancer stem cells
NEPC: Neuroendocrine prostate cancer
lncRNA: Long non-coding RNA
miRNA: Micro RNA
RMI2: RecQ-mediated genome instability 2
TOPBP1: DNA topoisomerase II binding protein 1
DNMT: DNA methyltransferase
LINE-1: Long interspersed nuclear element-1
TET: Ten-eleven translocation
APC: Adenomatous polyposis coli
TSG: Tumor supressor gene
RAR*β*2: Retinoic acid receptor beta2
RASSF1A: Ras association domain family member1A
CDNK2A: Cyclin-dependent kinase inhibitor2A
HOXD3: Homeobox gene D3
MGMT: O^6^-methylguanine DNA methyltransferase
PTGS2: Prostaglandin-endoperoxide synthase 2
CCND2: Cyclin2
GSTP1: Glutathione S-transferase Pi1
KMT: Lysine methyltransferase
KDM: Lysine demethylase
EZH2: Enhancer of zeste homolog 2
JmjC: Jumonji c domain
CBP: P300/CREB binding protein
HAT: Histone acetyltransferase
TNF: Tumor necrosis factor
NF-kB: Nuclear factor kappa-light-chain-enhancer of activated B cells
SIRT: Sirtuins
XAF1: XIAP-associated factor 1
YY1: *Yan Yang 1*
PTM: Post translational modifications
IR: Ionizing radiation
HDACi: HDAC inhibitors
DNMTi: DNMT inhibitors
