# Supplementary material for: Epigenetic mechanisms underlying prostate cancer radioresistance
Source: Clin Epigenetics. 2021 Jun 8;13:125. doi: 10.1186/s13148-021-01111-8 (PMC8186094; doi:10.1186/s13148-021-01111-8)
Supplement: Supplementary file 1 — Additional file 1. Complementary pre-clinical studies for tumor radiosensitization. [file 13148_2021_1111_MOESM1_ESM.docx]

**Macedo-Silva et al** **_Supplementary file 1**

This file comprises supplementary information concerning pre-clinical studies mentioned on figure 4. Specifically Vorinostat (SAHA), a well-known HDACi was described as a potent radiosensitizer [1-20]. Likewise, 15 pre-clinical studies demonstrated the relevance of SAHA concomitantly with conventional radiotherapeutic schemes for several cancer types including, prostate cancer, glioma, colorectal cancer, brain metastasis, osteosarcomas, rhabdomyosarcomas, neuroblastoma, multiple myeloma, lung metastasis, acute myeloid leukemia, cervical cancer, non-small cell lung cancer, pancreatic cancer, glioblastoma and advanced head and neck cancer.

In the same vein, valproic acid (VPA) [21-35], trichostatin A (TSA) [36-41], panobinostat (LBH-589) [42-46] and entinostat (MS-275) represented a considerable number of 20 pre-clinical studies with radiosensitization purpose for leukemia, colorectal cancer, retinoblastoma, prostate cancer, glioma, esophageal squamous cell carcinoma, non-small cell lung cancer, glioblastoma multiforme, breast cancer, cervical cancer, tongue squamous cell carcinoma and bladder cancer.

To highlight, all previous mentioned radiosensitizers HDACi were also evaluated in prostate cancer. Otherwise, other HDACi such as, FK228, CBHA, sodium butyrate, TMP196 and MGCD0103 were only reported in gastric and colorectal cancer, esophageal squamous cell carcinoma, thyroid cancer and hypoxic bladder cancer [47-58].

Furthermore, the synergistic effect of both HDACi and DNMTi involves also an important research on radiosensitization field. Indeed, 5-Aza-2’-deoxycytidine (DAC) and hydralazine in combination with other previous mentioned HDACi were reported in colorectal and breast cancer, cervical cancer and medulloblastoma [59-62]. The effect of DAC to improve radiotherapeutic efficacy rates was still studied with promising results in gastric cancer, medulloblastoma, breast cancer, osteosarcoma, colorectal cancer nasopharyngeal carcinoma, head and neck and laryngeal squamous cell carcinoma [48, 50, 51, 62-65].

Concerning histone methylation erasers, only few recent studies evolving JmjC-KDMs subfamily inhibitors, like GSK-J4 [66-68] and JIB-04 [66-70] were reported with a radiosensitizer effect in malignant pleural mesothelioma, lung squamous cell carcinoma, breast cancer, glioblastoma and diffuse intrinsic pontine glioma.

**References**

[1] Chinnaiyan P, Vallabhaneni G, Armstrong E, Huang SM, Harari PM. Modulation of radiation response by histone deacetylase inhibition. International journal of radiation oncology, biology, physics. 2005;62:223-9.

[2] Folkvord S, Ree AH, Furre T, Halvorsen T, Flatmark K. Radiosensitization by SAHA in experimental colorectal carcinoma models-in vivo effects and relevance of histone acetylation status. International journal of radiation oncology, biology, physics. 2009;74:546-52.

[3] Baschnagel A, Russo A, Burgan WE, Carter D, Beam K, Palmieri D, et al. Vorinostat enhances the radiosensitivity of a breast cancer brain metastatic cell line grown in vitro and as intracranial xenografts. Molecular cancer therapeutics. 2009;8:1589-95.

[4] Blattmann C, Oertel S, Ehemann V, Thiemann M, Huber PE, Bischof M, et al. Enhancement of radiation response in osteosarcoma and rhabdomyosarcoma cell lines by histone deacetylase inhibition. International journal of radiation oncology, biology, physics. 2010;78:237-45.

[5] Mueller S, Yang X, Sottero TL, Gragg A, Prasad G, Polley MY, et al. Cooperation of the HDAC inhibitor vorinostat and radiation in metastatic neuroblastoma: efficacy and underlying mechanisms. Cancer Lett. 2011;306:223-9.

[6] Chen X, Wong P, Radany EH, Stark JM, Laulier C, Wong JY. Suberoylanilide hydroxamic acid as a radiosensitizer through modulation of RAD51 protein and inhibition of homology-directed repair in multiple myeloma. Molecular cancer research : MCR. 2012;10:1052-64.

[7] Saelen MG, Ree AH, Kristian A, Fleten KG, Furre T, Hektoen HH, et al. Radiosensitization by the histone deacetylase inhibitor vorinostat under hypoxia and with capecitabine in experimental colorectal carcinoma. Radiation oncology (London, England). 2012;7:165.

[8] Chiu HW, Yeh YL, Wang YC, Huang WJ, Chen YA, Chiou YS, et al. Suberoylanilide hydroxamic acid, an inhibitor of histone deacetylase, enhances radiosensitivity and suppresses lung metastasis in breast cancer in vitro and in vivo. PLoS One. 2013;8:e76340.

[9] Blattmann C, Thiemann M, Stenzinger A, Christmann A, Roth E, Ehemann V, et al. Radiosensitization by histone deacetylase inhibition in an osteosarcoma mouse model. Strahlenther Onkol. 2013;189:957-66.

[10] Chen X, Radany EH, Wong P, Ma S, Wu K, Wang B, et al. Suberoylanilide hydroxamic acid induces hypersensitivity to radiation therapy in acute myelogenous leukemia cells expressing constitutively active FLT3 mutants. PLoS One. 2013;8:e84515.

[11] Ree AH, Saelen MG, Kalanxhi E, Østensen IH, Schee K, Røe K, et al. Biomarkers of histone deacetylase inhibitor activity in a phase 1 combined-modality study with radiotherapy. PLoS One. 2014;9:e89750.

[12] Shi W, Lawrence YR, Choy H, Werner-Wasik M, Andrews DW, Evans JJ, et al. Vorinostat as a radiosensitizer for brain metastasis: a phase I clinical trial. J Neurooncol. 2014;118:313-9.

[13] Xing J, Wang H, Xu S, Han P, Xin M, Zhou JL. Sensitization of suberoylanilide hydroxamic acid (SAHA) on chemoradiation for human cervical cancer cells and its mechanism. Eur J Gynaecol Oncol. 2015;36:117-22.

[14] Feng J, Zhang S, Wu K, Wang B, Wong JY, Jiang H, et al. Combined Effects of Suberoylanilide Hydroxamic Acid and Cisplatin on Radiation Sensitivity and Cancer Cell Invasion in Non-Small Cell Lung Cancer. Molecular cancer therapeutics. 2016;15:842-53.

[15] Jonsson M, Ragnum HB, Julin CH, Yeramian A, Clancy T, Frikstad KM, et al. Hypoxia-independent gene expression signature associated with radiosensitisation of prostate cancer cell lines by histone deacetylase inhibition. Br J Cancer. 2016;115:929-39.

[16] Wu Z, Jing S, Li Y, Gao Y, Yu S, Li Z, et al. The effects of SAHA on radiosensitivity in pancreatic cancer cells by inducing apoptosis and targeting RAD51. Biomed Pharmacother. 2017;89:705-10.

[17] Choi CYH, Wakelee HA, Neal JW, Pinder-Schenck MC, Yu HM, Chang SD, et al. Vorinostat and Concurrent Stereotactic Radiosurgery for Non-Small Cell Lung Cancer Brain Metastases: A Phase 1 Dose Escalation Trial. Int J Radiat Oncol Biol Phys. 2017;99:16-21.

[18] Gerelchuluun A, Maeda J, Manabe E, Brents CA, Sakae T, Fujimori A, et al. Histone Deacetylase Inhibitor Induced Radiation Sensitization Effects on Human Cancer Cells after Photon and Hadron Radiation Exposure. Int J Mol Sci. 2018;19.

[19] Galanis E, Anderson SK, Miller CR, Sarkaria JN, Jaeckle K, Buckner JC, et al. Phase I/II trial of vorinostat combined with temozolomide and radiation therapy for newly diagnosed glioblastoma: results of Alliance N0874/ABTC 02. Neuro Oncol. 2018;20:546-56.

[20] Teknos TN, Grecula J, Agrawal A, Old MO, Ozer E, Carrau R, et al. A phase 1 trial of Vorinostat in combination with concurrent chemoradiation therapy in the treatment of advanced staged head and neck squamous cell carcinoma. Invest New Drugs. 2019;37:702-10.

[21] Rezacova M, Zaskodova D, Vavrova J, Vokurkova D, Tichy A. Antileukemic activity of the combination of ionizing radiation with valproic acid in promyelocytic leukemia cells HL-60. Neoplasma. 2008;55:519-25.

[22] Chen X, Wong P, Radany E, Wong JY. HDAC inhibitor, valproic acid, induces p53-dependent radiosensitization of colon cancer cells. Cancer Biother Radiopharm. 2009;24:689-99.

[23] Kawano T, Akiyama M, Agawa-Ohta M, Mikami-Terao Y, Iwase S, Yanagisawa T, et al. Histone deacetylase inhibitors valproic acid and depsipeptide sensitize retinoblastoma cells to radiotherapy by increasing H2AX phosphorylation and p53 acetylation-phosphorylation. Int J Oncol. 2010;37:787-95.

[24] Van Nifterik KA, Van den Berg J, Slotman BJ, Lafleur MV, Sminia P, Stalpers LJ. Valproic acid sensitizes human glioma cells for temozolomide and γ-radiation. J Neurooncol. 2012;107:61-7.

[25] Shao CJ, Wu MW, Chen FR, Li C, Xia YF, Chen ZP. Histone deacetylase inhibitor, 2-propylpentanoic acid, increases the chemosensitivity and radiosensitivity of human glioma cell lines in vitro. Chin Med J (Engl). 2012;125:4338-43.

[26] Shoji M, Ninomiya I, Makino I, Kinoshita J, Nakamura K, Oyama K, et al. Valproic acid, a histone deacetylase inhibitor, enhances radiosensitivity in esophageal squamous cell carcinoma. Int J Oncol. 2012;40:2140-6.

[27] Zhou Y, Xu Y, Wang H, Niu J, Hou H, Jiang Y. Histone deacetylase inhibitor, valproic acid, radiosensitizes the C6 glioma cell line in vitro. Oncol Lett. 2014;7:203-8.

[28] Gavrilov V, Lavrenkov K, Ariad S, Shany S. Sodium valproate, a histone deacetylase inhibitor, enhances the efficacy of vinorelbine-cisplatin-based chemoradiation in non-small cell lung cancer cells. Anticancer research. 2014;34:6565-72.

[29] Hosein AN, Lim YC, Day B, Stringer B, Rose S, Head R, et al. The effect of valproic acid in combination with irradiation and temozolomide on primary human glioblastoma cells. J Neurooncol. 2015;122:263-71.

[30] Krauze AV, Myrehaug SD, Chang MG, Holdford DJ, Smith S, Shih J, et al. A Phase 2 Study of Concurrent Radiation Therapy, Temozolomide, and the Histone Deacetylase Inhibitor Valproic Acid for Patients With Glioblastoma. Int J Radiat Oncol Biol Phys. 2015;92:986-92.

[31] Makita N, Ninomiya I, Tsukada T, Okamoto K, Harada S, Nakanuma S, et al. Inhibitory effects of valproic acid in DNA double-strand break repair after irradiation in esophageal squamous carcinoma cells. Oncol Rep. 2015;34:1185-92.

[32] Choo DW, Goh SH, Cho YW, Baek HJ, Park EJ, Motoyama N, et al. CHK2 is involved in the p53-independent radiosensitizing effects of valproic acid. Oncol Lett. 2017;13:2591-8.

[33] Chie EK, Shin JH, Kim JH, Kim HJ, Kim IA, Kim IH. In Vitro and In Vivo Radiosensitizing Effect of Valproic Acid on Fractionated Irradiation. Cancer Res Treat. 2015;47:527-33.

[34] Chen X, Wong JY, Wong P, Radany EH. Low-dose valproic acid enhances radiosensitivity of prostate cancer through acetylated p53-dependent modulation of mitochondrial membrane potential and apoptosis. Mol Cancer Res. 2011;9:448-61.

[35] Karagiannis TC, Kn H, El-Osta A. The epigenetic modifier, valproic acid, enhances radiation sensitivity. Epigenetics. 2006;1:131-7.

[36] Kim JH, Shin JH, Kim IH. Susceptibility and radiosensitization of human glioblastoma cells to trichostatin A, a histone deacetylase inhibitor. International journal of radiation oncology, biology, physics. 2004;59:1174-80.

[37] Zhang F, Zhang T, Teng ZH, Zhang R, Wang JB, Mei QB. Sensitization to gamma-irradiation-induced cell cycle arrest and apoptosis by the histone deacetylase inhibitor trichostatin A in non-small cell lung cancer (NSCLC) cells. Cancer Biol Ther. 2009;8:823-31.

[38] Yu J, Mi J, Wang Y, Wang A, Tian X. Regulation of radiosensitivity by HDAC inhibitor trichostatin A in the human cervical carcinoma cell line Hela. Eur J Gynaecol Oncol. 2012;33:285-90.

[39] Frame FM, Pellacani D, Collins AT, Simms MS, Mann VM, Jones GD, et al. HDAC inhibitor confers radiosensitivity to prostate stem-like cells. Br J Cancer. 2013;109:3023-33.

[40] He G, Wang Y, Pang X, Zhang B. Inhibition of autophagy induced by TSA sensitizes colon cancer cell to radiation. Tumour Biol. 2014;35:1003-11.

[41] Jia L, Zhang S, Huang Y, Zheng Y, Gan Y. Trichostatin A increases radiosensitization of tongue squamous cell carcinoma via miR‑375. Oncol Rep. 2017;37:305-12.

[42] Groselj B, Kerr M, Kiltie AE. Radiosensitisation of bladder cancer cells by panobinostat is modulated by Ku80 expression. Radiother Oncol. 2013;108:429-33.

[43] Xiao W, Graham PH, Hao J, Chang L, Ni J, Power CA, et al. Combination therapy with the histone deacetylase inhibitor LBH589 and radiation is an effective regimen for prostate cancer cells. PLoS One. 2013;8:e74253.

[44] Takhar HS, Singhal N, Gowda R, Penniment M, Takhar P, Brown MP. Phase I study evaluating the safety and efficacy of oral panobinostat in combination with radiotherapy or chemoradiotherapy in patients with inoperable stage III non-small-cell lung cancer. Anticancer Drugs. 2015;26:1069-77.

[45] Shi W, Palmer JD, Werner-Wasik M, Andrews DW, Evans JJ, Glass J, et al. Phase I trial of panobinostat and fractionated stereotactic re-irradiation therapy for recurrent high grade gliomas. J Neurooncol. 2016;127:535-9.

[46] Nicholson J, Jevons SJ, Groselj B, Ellermann S, Konietzny R, Kerr M, et al. E3 Ligase cIAP2 Mediates Downregulation of MRE11 and Radiosensitization in Response to HDAC Inhibition in Bladder Cancer. Cancer Res. 2017;77:3027-39.

[47] Patties I, Jahns J, Hildebrandt G, Kortmann RD, Glasow A. Additive effects of 5-aza-2'-deoxycytidine and irradiation on clonogenic survival of human medulloblastoma cell lines. Strahlenther Onkol. 2009;185:331-8.

[48] Kim JG, Bae JH, Kim JA, Heo K, Yang K, Yi JM. Combination effect of epigenetic regulation and ionizing radiation in colorectal cancer cells. PLoS One. 2014;9:e105405.

[49] Wang S, Zhang R, Claret FX, Yang H. Involvement of microRNA-24 and DNA methylation in resistance of nasopharyngeal carcinoma to ionizing radiation. Molecular cancer therapeutics. 2014;13:3163-74.

[50] Chen X, Liu L, Mims J, Punska EC, Williams KE, Zhao W, et al. Analysis of DNA methylation and gene expression in radiation-resistant head and neck tumors. Epigenetics. 2015;10:545-61.

[51] Kim JS, Kim SY, Lee M, Kim SH, Kim SM, Kim EJ. Radioresistance in a human laryngeal squamous cell carcinoma cell line is associated with DNA methylation changes and topoisomerase II α. Cancer Biol Ther. 2015;16:558-66.

[52] Hofstetter B, Niemierko A, Forrer C, Benhattar J, Albertini V, Pruschy M, et al. Impact of genomic methylation on radiation sensitivity of colorectal carcinoma. International journal of radiation oncology, biology, physics. 2010;76:1512-9.

[53] Wee CW, Kim JH, Kim HJ, Kang HC, Suh SY, Shin BS, et al. Radiosensitization of Glioblastoma Cells by a Novel DNA Methyltransferase-inhibiting Phthalimido-Alkanamide Derivative. Anticancer research. 2019;39:759-69.

[54] Kim HJ, Kim JH, Chie EK, Young PD, Kim IA, Kim IH. DNMT (DNA methyltransferase) inhibitors radiosensitize human cancer cells by suppressing DNA repair activity. Radiation oncology (London, England). 2012;7:39.

[55] Zhang Y, Adachi M, Zhao X, Kawamura R, Imai K. Histone deacetylase inhibitors FK228, N-(2-aminophenyl)-4-[N-(pyridin-3-yl-methoxycarbonyl)amino- methyl]benzamide and m-carboxycinnamic acid bis-hydroxamide augment radiation-induced cell death in gastrointestinal adenocarcinoma cells. Int J Cancer. 2004;110:301-8.

[56] Dong Q, Sharma S, Liu H, Chen L, Gu B, Sun X, et al. HDAC inhibitors reverse acquired radio resistance of KYSE-150R esophageal carcinoma cells by modulating Bmi-1 expression. Toxicol Lett. 2014;224:121-9.

[57] Perona M, Thomasz L, Rossich L, Rodriguez C, Pisarev MA, Rosemblit C, et al. Radiosensitivity enhancement of human thyroid carcinoma cells by the inhibitors of histone deacetylase sodium butyrate and valproic acid. Mol Cell Endocrinol. 2018;478:141-50.

[58] Groselj B, Ruan JL, Scott H, Gorrill J, Nicholson J, Kelly J, et al. Radiosensitization In Vivo by Histone Deacetylase Inhibition with No Increase in Early Normal Tissue Radiation Toxicity. Mol Cancer Ther. 2018;17:381-92.

[59] Cho HJ, Kim SY, Kim KH, Kang WK, Kim JI, Oh ST, et al. The combination effect of sodium butyrate and 5-Aza-2'-deoxycytidine on radiosensitivity in RKO colorectal cancer and MCF-7 breast cancer cell lines. World J Surg Oncol. 2009;7:49.

[60] Candelaria M, Cetina L, Pérez-Cárdenas E, de la Cruz-Hernández E, González-Fierro A, Trejo-Becerril C, et al. Epigenetic therapy and cisplatin chemoradiation in FIGO stage IIIB cervical cancer. Eur J Gynaecol Oncol. 2010;31:386-91.

[61] Mani E, Medina LA, Isaac-Olivé K, Dueñas-González A. Radiosensitization of cervical cancer cells with epigenetic drugs hydralazine and valproate. Eur J Gynaecol Oncol. 2014;35:140-2.

[62] Patties I, Kortmann RD, Menzel F, Glasow A. Enhanced inhibition of clonogenic survival of human medulloblastoma cells by multimodal treatment with ionizing irradiation, epigenetic modifiers, and differentiation-inducing drugs. J Exp Clin Cancer Res. 2016;35:94.

[63] Qiu H, Yashiro M, Shinto O, Matsuzaki T, Hirakawa K. DNA methyltransferase inhibitor 5-aza-CdR enhances the radiosensitivity of gastric cancer cells. Cancer Sci. 2009;100:181-8.

[64] Wang L, Zhang Y, Li R, Chen Y, Pan X, Li G, et al. 5-aza-2'-Deoxycytidine enhances the radiosensitivity of breast cancer cells. Cancer Biother Radiopharm. 2013;28:34-44.

[65] Li Y, Geng P, Jiang W, Wang Y, Yao J, Lin X, et al. Enhancement of radiosensitivity by 5-Aza-CdR through activation of G2/M checkpoint response and apoptosis in osteosarcoma cells. Tumour Biol. 2014;35:4831-9.

[66] Cregan S, Breslin M, Roche G, Wennstedt S, MacDonagh L, Albadri C, et al. Kdm6a and Kdm6b: Altered expression in malignant pleural mesothelioma. Int J Oncol. 2017;50:1044-52.

[67] Rath BH, Waung I, Camphausen K, Tofilon PJ. Inhibition of the Histone H3K27 Demethylase UTX Enhances Tumor Cell Radiosensitivity. Molecular cancer therapeutics. 2018;17:1070-8.

[68] Katagi H, Louis N, Unruh D, Sasaki T, He X, Zhang A, et al. Radiosensitization by Histone H3 Demethylase Inhibition in Diffuse Intrinsic Pontine Glioma. Clinical cancer research : an official journal of the American Association for Cancer Research. 2019;25:5572-83.

[69] Bayo J, Tran TA, Wang L, Peña-Llopis S, Das AK, Martinez ED. Jumonji Inhibitors Overcome Radioresistance in Cancer through Changes in H3K4 Methylation at Double-Strand Breaks. Cell Rep. 2018;25:1040-50.e5.

[70] Pippa S, Mannironi C, Licursi V, Bombardi L, Colotti G, Cundari E, et al. Small Molecule Inhibitors of KDM5 Histone Demethylases Increase the Radiosensitivity of Breast Cancer Cells Overexpressing JARID1B. Molecules. 2019;24.
